# Asthma control in patients receiving inhaled corticosteroid and long-acting beta_2_-agonist fixed combinations. A real-life study comparing dry powder inhalers and a pressurized metered dose inhaler extrafine formulation

**DOI:** 10.1186/1471-2466-11-40

**Published:** 2011-07-15

**Authors:** Veronika Müller, Gabriella Gálffy, Noemi Eszes, György Losonczy, Andrea Bizzi, Gabriele Nicolini, Henry Chrystyn, Lilla Tamási

**Affiliations:** 1 Department of Pulmonology, Semmelweis Medical University, Budapest, Hungary; 2 Chiesi Farmaceutici S.p.A., Parma, Italy; 3 Division of Pharmacy, School of Applied Sciences, University of Huddersfield, Queensgate, Huddersfield, West Yorkshire, HD1 3DH, UK

**Keywords:** inhaler, fixed combinations, asthma control, extrafine

## Abstract

**Background:**

Although patients have more problems using metered dose inhalers, clinical comparisons suggest they provide similar control to dry powder inhalers. Using real-life situations this study was designed to evaluate asthma control in outpatients with moderate to severe persistent asthma and to compare efficacy of fixed combinations of inhaled corticosteroids (ICS) and long acting beta-agonists (LABA).

**Methods:**

This real-life study had a cross-sectional design. Patients using fixed combinations of ICS and LABA had their asthma control and spirometry assessed during regular visits.

**Results:**

111 patients were analyzed: 53 (47.7%) received maintenance therapy of extrafine beclomethasone-formoterol (BDP/F) pressurized metered dose inhaler (pMDI), 25 (22.5%) fluticasone-salmeterol (FP/S) dry powder inhaler (DPI), and 33 (29.7%) budesonide-formoterol (BUD/F) DPI. Severity of asthma at time of diagnosis, assessed by the treating physician, was comparable among groups. Asthma control was achieved by 45.9% of patients; 38.7% were partially controlled and 15.3% were uncontrolled. In the extrafine BDF/F group, asthma control total score, daytime symptom score and rescue medication use score were significantly better than those using fixed DPI combinations (5.8 ± 6.2 vs. 8.5 ± 6.8; 1.4 ± 1.8 vs. 2.3 ± 2.1; 1.8 ± 2.2 vs. 2.6 ± 2.2; p = 0.0160; p = 0.012 and p = 0.025, respectively) and the mean daily ICS dose were significantly lower.

**Conclusions:**

pMDI extrafine BDP/F combination demonstrated better asthma control compared to DPIs formulated with larger particles. This could be due to the improved lung deposition of the dose or less reliance on the optimal inhalation technique or both.

## Background

While asthma represents a global public health issue due to high prevalence rates in the general population (ranging from 1% to 18% of the population in different Countries), several studies now indicate that the impact on public health is even more severe due to the difficulty in achieving full disease control with available therapies [1,2]. Indeed, achieving and maintaining asthma control is the major goal of asthma care. Based on the Global Initiative for Asthma (GINA) 2009 guidelines, asthma control can be assessed by taking into consideration different characteristics including daytime symptoms, nocturnal awakenings, need of rescue medication, limitation of physical activity, and lung function [2]. Randomized clinical trials (RCTs) have shown that current therapeutic asthma treatments are efficient in granting good asthma control [3]. However, efficacy results reported in RCTs are often inconsistent with data observed in real-life settings where asthma control still remains an unmet need for the great majority of patients [1,4]. Despite the variety of treatment options available and the periodical update of GINA guidelines since 1995, the evidence indicates suboptimal asthma control, even if improvements are evident when comparing more recent data [1] with previous ones [5], in a great number of patients.

However, RCTs are performed under highly controlled settings and in selected populations of patients. Under these controlled conditions, good inhalation technique is usually checked and granted and the importance of compliance is emphasized. Such a level of adherence is not to be expected in real-life settings. In an effort to find a strategy leading to improved asthma care, the International Primary Care Respiratory Group (IPCRG) examined the main factors contributing to poor control of the disease [6]. Among the several identified reasons, wrong diagnosis, smoking, co-morbidities, individual variation in response to treatment, poor adherence, and poor inhalation technique have been described.

Inhalers for fixed combinations of ICS and LABA include pMDIs and DPIs. It is known that choice of the correct inhaler device is crucial since sub-optimal inhalation techniques can result in reduced drug delivery and efficacy and it should be adjusted to different patients [7,8]. A meta-analysis of RCTs comparing the same drug delivered by different devices concluded that efficacy outcomes do not differ significantly and that pMDIs and DPIs are equally efficacious in clinical settings [9,10], although studies have shown that there are fewer inhalation errors with some DPIs [11-13].

A new fixed combination of BDP/F has been recently developed. The innovative formulation of BDP/F pMDI is characterized by extrafine particle size [14] which results in improved lung deposition and allows for uniform treatment of inflammation and bronchoconstriction throughout the entire bronchial tree [15]. This can translate into beneficial asthma outcomes as demonstrated by a significant increase in asthma control vs. a combination of the same drugs (BDP plus F) given as larger (not extrafine) particles [16]. It has been reported that the extrafine formulation of BDP/F allows for a longer duration of the aerosol plume and a reduced need for hand-to-lung coordination, thus facilitating patient inhaler technique [17]. Furthermore, it has been suggested that pMDI inhalation technique for formulations that produce extrafine particles is less critical than with other MDIs [8], because lung deposition is less affected by inhalation flow [18] and coordination [19]. Moreover, the fast onset of bronchodilation may improve patient adherence [20] and contribute to efficacy.

The aim of this observational study was to evaluate the level of asthma control in a group of patients with moderate or severe persistent asthma (at the time of diagnosis) and to compare, in a real-life setting, efficacy outcomes achieved with extrafine BDP/F delivered via pMDI vs. ICS-LABA combinations (FP/S and BUD/F) delivered via DPIs.

## Methods

### Study design and patients

This real-life study had a cross-sectional design and involved a group of asthmatic outpatients from the Department of Pulmonology, Semmelweis University Budapest, Hungary. Patients were consecutively recruited during their regular visits to the outpatient department from May 2008 until August 2008.

Male and female asthma patients aged above 18 years were eligible for inclusion in the study if: 1) they had a diagnosis of moderate or severe persistent asthma confirmed by chest physician; 2) diagnosis occurred at least 6 months before the beginning of the study; 3) they were treated with fixed combination of ICS and LABA as maintenance therapy; 4) they did not change their medication within the last 4 weeks prior to start of study; 5) they had no asthmatic exacerbation within the last 6 weeks prior to start of study. Patients were excluded if they had any other chronic disease (except chronic rhinitis), extreme obesity, or if any acute disease occurred within the last 6 weeks prior to start of study. Patients were enrolled regardless of their smoking history or habit. Asthma severity was evaluated at the time of diagnosis and assessed by the treating physician.

The study was approved by the institutional Ethics Committee of the Department of Pulmonology, Semmelweis University. No consent was obtained from the participants as it was a non-interventional retrospective data analysis of the real-life data collected on the usual visits of patients. No intervention was done only for the sake of the study.

### Assessment of asthma control

Asthma control was assessed by patients using a questionnaire (Table 1) developed on the basis of the GINA Guidelines. A score ≤ 4 indicated well controlled asthma, a score >14 reflected uncontrolled asthma, and intermediate scores were indicative of partially controlled asthma. Patients were asked to fill in the questionnaire during their regular clinic visits.

**Table 1:** Questionnaire for the assessment of asthma control

1. **Have daytime asthma symptoms occurred during the last week (dyspnoea, shortness of breath, wheezing)?**	
a. No	0 points
b. Yes, maximum twice a week	1 point
c. Yes, more than twice a week	5 points

2. **Have night-time symptoms occurred OR have you been woken up by asthma symptoms during the last week?**	

a. No	0 points
b. Yes, but I am not sure if it was asthma	1 point
c. Yes	5 points

3. **Have you felt any limitation during physical activity during the last week?**	

a. No	0 points
b. Yes, but only during hard physical work	1 point
c. Yes, during normal daily activities	5 points

4. How frequently have you used reliever therapy during the last week(Ventolin/Berotec/Berodual/Salbutamol/Atrovent/Bricanyl/Symbicort reliever)?*	

a. Never	0 points
b. Twice or less	1 point
c. More than twice	5 points

5. **Have you had an acute exacerbation/attack of asthma that resulted in emergency department/hospitalization since you started using this maintenance therapy?**	

a. No	0 points
b. Yes	5 points

**Total score:**	

CONTROLLED ASTHMA = 0-4 points	
PARTIALLY CONTROLLED ASTHMA = 5-14 points	
UNCONTROLLED ASTHMA = more than 14	

* Ventolin/Berotec/Berodual/Salbutamol/Atrovent were all MDI formulations.

To determine if other factors may influence the level of asthma control, the following information was collected from patient records: date of diagnosis, smoking status, history of allergy, asthma maintenance and reliever therapy.

Spirometric measurements were performed and data on forced expiratory volume at 1 s (FEV_1_), peak expiratory flow (PEF), forced expiratory flow between 25 and 75% (FEF 25-75%), maximum expiratory flow (MEF) 25%, and MEF 75% were collected. Lung function was analyzed using an electronic spirometer (PDD-301/s, Piston, Budapest, Hungary) according to the American Thoracic Society (ATS) guidelines [21].

### Statistical Analysis

Data were analyzed with the MEDCALC statistical program package. Normal distribution was checked and tested formally by the Kolmogorov-Smirnov test. Differences between groups were tested by parametric or nonparametric tests as appropriate. The Student t test was used to test differences in case of normal distribution between the groups. Kruskal-Wallis and Mann-Whitney U tests were used for statistical comparison of the groups in case of non-normal distribution. Chi-square test was used where indicated. The level of significance was set at 0.05.

## Results

### Clinical data of the patients

The study included 111 patients (81 women and 30 men). For the purpose of the study, patients were divided in two groups depending on the inhaler device they were using (pMDI or DPI). No significant difference in patient characteristics between these two groups was found (Table 2). The severity of patients was similar in both groups. Lung function values measured at the time of diagnosis, on the basis of which patients were characterized as moderate or severe asthmatics, were not available. Lung function values that are indicated in Table 2 were measured during patient visits (using regular asthma maintenance therapy). No significant difference was detected between the two treatment groups in terms of spirometric values.

**Table 2:** Clinical characteristics and lung function parameters in different treatment groups

	BDP/F extrafine pMDI (n = 53)	BUD/F and FP/S DPI (n = 58)
Male, n (%)	15 (28.3%)	15(25.8%)

Age, years (mean and range)	48 (19-84)	47 (18-86)

Current smokers, n (%)	20 (37.7%)	19 (32.8%)

Allergies, n (%)	40 (75.5%)	41 (70.6%)

Patients using SABA for as needed medication, n (%)	53 (100%)	50 (86.2%)

Patients using BUD/F for as needed medication, n (%)	0 (0%)	8 (13.8%)

Initial diagnosis of mild persistent asthma, n (%)	0 (0%)	0 (0%)

Initial diagnosis of moderate persistent asthma, n (%)	45 (84.9%)	49 (84.5%)

Initial diagnosis of severe persistent asthma, n (%)	8 (15.1%)	9 (15.5%)

FEV_1_(mean ± SE; % of predicted)	81.71 ± 17.53	79.38 ± 17.71

PEF (mean ± SE; % of predicted)	78.47 ± 19.92	75.34 ± 22.12

FEF _25-75 _(mean ± SE; % of predicted)	62.35 ± 26.18	56.52 ± 23.3

MEF _75 _(mean ± SE; % of predicted)	71.53 ± 28.38	71.41 ± 29.01

MEF _25 _(mean ± SE; % of predicted)	58.2 ± 26.12	53.74 ± 24.23

SABA = short acting beta_2_-agonist; BDP = beclomethasone dipropionate; BUD = budesonide; FP = fluticasone propionate; F = formoterol; S = salmeterol. ^**#**^In pMDI group spirometry data were not available in 2 patients.

### Asthma control results

**Figure 1:**
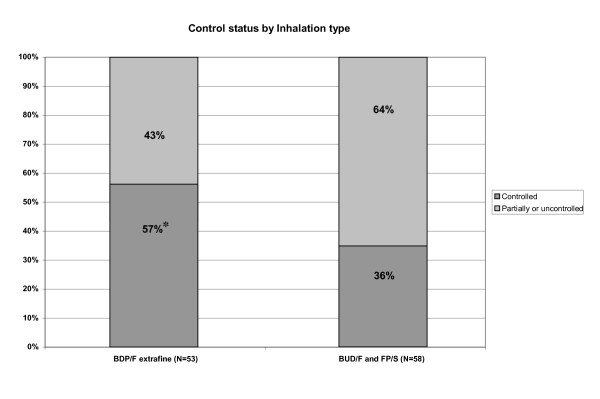
Proportion of patients with different levels of asthma control in the two study groups; *p = 0.031 pMDI vs. BUD/F and FP/S. BDP-beclomethasone dipropionate; BUD- budesonide; FP-fluticasone propionate; F-formoterol; S-salmeterol.

The proportions of patients with different levels of asthma control in the two study groups and in the whole study population are shown in Table 3 and on Figure 1. Comparing the two device types, a significantly higher proportion of controlled patients was observed in the pMDI group when compared to DPI group (p = 0.031; Chi-square test)

**Table 3:** Proportion of patients with different levels of asthma control

	BDP/F extrafine pMDI (n = 53)	BUD/F and FP/S DPI (n = 58)	All (n = 111)
Controlled	30 (56.60%)*	21 (36.21%)	51 (45.94%)

Partially controlled	17 (32.08%)	26 (44.83%)	43 (38.74%)

Uncontrolled	6 (11.32%)	11 (18.96%)	17 (15.32%)

BDP = beclomethasone dipropionate; BUD = budesonide; FP = fluticasone propionate; F = formoterol; S = salmeterol.* p = 0.031 vs. BUD/F and FP/S.

**Figure 2:**
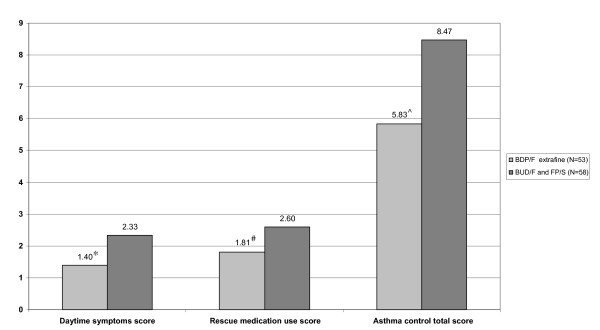
Daytime symptom score, rescue medication use score and asthma control total score achieved by BDP/F extrafine pMDI or BUD/F and FP/S DPI; *p = 0.012, ^#^p = 0.025, and ^^^p = 0.016, respectively (Mann-Whitney Test for BDP/F vs. BUD/F and FP/S). BDP-beclomethasone dipropionate; BUD- budesonide; FP-fluticasone propionate; F-formoterol; S-salmeterol.

Mean asthma control score was significantly lower for the extrafine pMDI group as compared to the DPI group (p = 0.016) indicating a better asthma control in extrafine pMDI-treated patients (Figure 2). When looking at single domains of asthma control, mean daytime symptom score was significantly lower in the extrafine pMDI group as compared to the DPI group (p = 0.012), demonstrating better symptom control in patients using extrafine pMDI (Figure 2). The same was true for rescue medication use that was significantly lower in the extrafine pMDI group (p = 0.025; Figure 2). Scores representing night-time symptoms and physical activity limitation did not differ significantly between the two groups.

**Figure 3:**
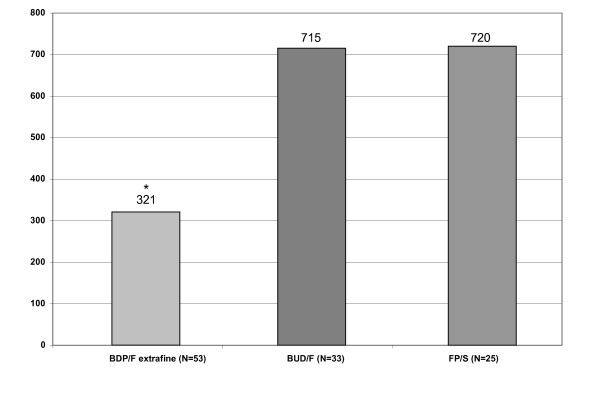
**Mean daily ICS doses (μg) in the different groups of patients**. *p < 0.0001 BDP/F vs. BUD/F and FP/S. BDP-beclomethasone dipropionate; BUD- budesonide; FP-fluticasone propionate; F-formoterol; S-salmeterol.

Notably, the mean daily ICS dose was significantly lower in the extrafine pMDI group as compared to the DPI group, suggesting an overall better asthma control with a lower steroid load (p < 0.001; Figure 3).

Asthma control levels detected by the questionnaire adopted in this study were in good agreement with the control levels assessed by using the current GINA guidelines. According to GINA-based control assessment (that includes lung function values as well), 38.74% of the patients proved to be controlled, 38.74% and 22.52% partially controlled and uncontrolled, respectively.

### Subgroup of smoking patients

Current or previous smoking was common in asthmatic patients enrolled in this study (35%), with a mean smoking history of 19.0 ± 3.6 pack/years. No gender difference between smokers was noted. While smoking status did not influence steroid dose used, it significantly increased the proportion of uncontrolled patients as compared to never smokers (11% vs. 23%, p < 0.05). Asthma control in smokers is summarized in Table 4.

**Table 4:** Asthma control in smoker subgroups

	BDP/F extrafine pMDI (n = 20)	BUD/F and FP/S DPI (n = 19)	All (n = 39)
Controlled	12 (60%)	8 (42.1%)	20 (51.3)

Partially controlled	5 (25%)	5 (26.3%)	10 (25.6%)

Uncontrolled	3 (15%)	6 (31.6%)	9 (23.1%)

BDP = beclomethasone dipropionate; BUD = budesonide; FP = fluticasone propionate; F = formoterol; S = salmeterol.

## Discussion

This is the first real-life study comparing the clinical effects of BDP/F extrafine to other combinations. Previously published RCTs found that there were no differences between BDP/F extrafine pMDI and BUD/F or FP/S in terms of lung function, use of rescue medications, exacerbations and safety [22,23]. However, these studies do not reflect routine use, during which compliance or each patient's ability to use the device may influence the result. In addition to the improved lung deposition and distribution, there is less reliance on using an optimal inhalation technique for pMDIs formulated with extrafine particles [8]. Although these were not assessed in this study, the result would include these elements. Previous studies have shown that, when inhalers emitting large particles are used, the magnitude of inhalation technique errors for a pMDI and DPI are similar [12].

The results show that asthma control was significantly better in patients receiving BDP/F extrafine pMDI formulation compared to the DPI group. Daytime symptoms and use of rescue medications were significantly lower in the BDP/F extrafine pMDI group, further supporting a greater control of asthma in a real-life setting. Asthma is an inflammatory disorder affecting both central and peripheral airways [24,25], which justifies the clinical need for anti-asthmatic medication which is also able to reach peripheral airways. The inverse correlation on particle size and pulmonary deposition is well known [26]. Of the fixed combinations of ICS currently available on the market, BDP/F extrafine pMDI drug particles are approximately half the size in diameter compared to FP/S and BUD/F DPIs [17]. BDP/F extrafine pMDI is the only fixed combination which has been demonstrated to reach not only large, but also small airways when administered in asthmatic patients [15]. Accordingly, the improved drug delivery, which enables a more uniform treatment of inflammation and bronchoconstriction throughout the entire bronchial tree, contributes to the higher level of asthma control achieved with BDP/F extrafine combination in this study. In other words, extrafine BDP/F can reach and treat distal areas of the lung left untargeted by larger particle sized medications, and this translates into a greater clinical benefit for patients. A significantly superior asthma control with the BDP/F extrafine combination vs. the combination of the same drugs (BDP plus F) given as larger particles, non extrafine agents, has been found in a large 6-month RCT carried-out in asthmatic patients [16]. However, in this study, the definition of asthma control was established according to Zetterström [27] and was therefore different from the GINA guidelines, which is a limitation of our analysis. No differences in lung function parameters were observed among treatments. This finding is not surprising considering that the spirometric tests performed in this study do not provide comprehensive evaluation of the entire bronchial tree as they are unable to properly reflect small airway abnormalities.

In our real-life study, current or former smokers were not excluded since they represent a large proportion of asthmatic patients. Generally, the level of asthma control in smokers was similar to that in non smokers; however, the proportion of uncontrolled patients was significantly increased in these patients as compared to never smokers. Smoking asthmatics are mostly excluded from clinical trials, therefore only sparse data are available on asthma control in current or former smoker asthmatic patients.

Notably, these results were obtained with a lower mean daily ICS dose indicating that BDP/F extrafine delivers a greater efficacy per μg of steroid when compared to BUD/F and FP/S. This is of interest considering that reduced compliance in asthmatic patients is often due to steroid phobia, an issue that can be decreased by offering a medication with a low corticosteroid dose [28].

In the studied population, 45.9% of patients had controlled asthma, 38.7% were partially controlled, and 15.3% were uncontrolled. These findings are in agreement with recent observational trials focusing on asthma control. A study performed across 10 different European countries showed that asthma is suitably controlled in 45% of non-inhaled corticosteroid users and only in 15% of patients using ICS [1]. Similarly, a web-based survey to assess the prevalence of uncontrolled asthma in the United States reported that symptoms were successfully controlled in 45% of patients [29].

This real-life observational study shows that GINA-based asthma control is achieved in a good proportion of asthmatic patients treated with fixed combinations of ICS and LABA. Patients treated with BDP/F extrafine pMDI achieved a greater level of asthma control as compared to patients treated with FP/S or BUD/F larger particle combinations, suggesting that differences in inhaler devices and formulations can have an impact on important clinical outcomes and should be therefore taken into consideration when managing patients with asthma.

## Conclusions

In asthmatic patients with comparable disease severity, the use of extrafine BDP/F combination formulated in a pMDI demonstrated better asthma control than using DPI fixed dose combinations formulated with larger particles. This could be due to the improved lung deposition and distribution of the dose or less reliance on the optimal inhalation technique or both.

## Conflict of interest declaration

Henry Chrystyn has no shares in any pharmaceutical companies. He has received sponsorship to carry out studies, together with consultant arrangements with Almirall, AstraZeneca, Boehringer Ingelheim, Chiesi, GlaxoSmithKline, Innovata Biomed, Meda, Napp Pharmaceuticals, Orion, Teva, Truddell and UCB. Research sponsorship has also been received from grant awarding bodies (EPSRC and MRC).

Lilla Tamasi, Veronika Müller, Gabriella Galffy and György Losonczy have no shares in any pharmaceutical companies. All of them have consultant arrangements with AstraZeneca, Boehringer Ingelheim, Chiesi, GlaxoSmithKline, MSD, Pfizer and Nycomed.

Andrea Bizzi and Gabriele Nicolini are employees of Chiesi Farmaceutici S.p.A.

The study was conducted by Semmelweis Medical University, Department of Pulmonology, Budapest, Hungary, without financial support from any pharmaceutical company.

## Authors' contributions

VM, GG, TL participated in conception and design of the study, acquisition, analysis and interpretation of data, as well as in drafting of the manuscript. EN, LGY participated in study design, analysis and interpretation of data and have been involved in drafting of the manuscript. GN and AB participated in drafting and reviewing the manuscript. HC provided external advice and received regular updates. He participated in the design and analysis of the data as well as involvement with drafting of the paper. All authors read and approved the final manuscript.

## Pre-publication history

The pre-publication history for this paper can be accessed here:

http://www.biomedcentral.com/1471-2466/11/40/prepub

## References

[B1] Cazzoletti L Marcon A Janson C Corsico A Jarvis D Pin I Accordini S Almar E Bugiani M Carolei A Cerveri I Duran-Tauleria E Gislason D Gulsvik A Jõgi R Marinoni A Martínez-Moratalla J Vermeire P de Marco R Therapy and Health Economics Group of the European Community Respiratory Health Survey. Asthma control in Europe: a real-world evaluation based on an international population-based study J Allergy Clin Immunol 2007 120 6 1360 7 10.1016/j.jaci.2007.09.019 17981317

[B2] GINA (Global Initiative on Asthma) National Institute of Health National Heart Lung and Blood Institute Global Strategy for asthma management and prevention 2009 http://www.ginasthma.com

[B3] Bateman ED Boushey HA Bousquet J Busse WW Clark TJ Pauwels RA Pedersen SE GOAL Investigators Group Can guideline-defined asthma control be achieved? The Gaining Optimal Asthma ControL study Am J Respir Crit Care Med 2004 170 8 836 44 10.1164/rccm.200401-033OC 15256389

[B4] Holgate S Bisgaard H Bjermer L Haahtela T Haughney J Horne R McIvor A Palkonen S Price DB Thomas M Valovirta E Wahn U The Brussels Declaration: the need for change in asthma management Eur Respir J 2008 32 6 1433 42 10.1183/09031936.00053108 19043008

[B5] Rabe KF Adachi M Lai CK Soriano JB Vermeire PA Weiss KB Weiss ST Worldwide severity and control of asthma in children and adults: the global asthma insights and reality surveys J Allergy Clin Immunol 2004 114 1 40 7 10.1016/j.jaci.2004.04.042 15241342

[B6] Haughney J Price D Kaplan A Chrystyn H Horne R May N Moffat M Versnel J Shanahan ER Hillyer EV Tunsäter A Bjermer L Achieving asthma control in practice: understanding the reasons for poor control Respir Med 2008 102 12 1681 93 10.1016/j.rmed.2008.08.003 18815019

[B7] Lavorini F Magnan A Dubus JC Voshaar T Corbetta L Broeders M Dekhuijzen R Sanchis J Viejo JL Barnes P Corrigan C Levy M Crompton GK Effect of incorrect use of dry powder inhalers on management of patients with asthma Respir Med 2008 102 4 593 604 10.1016/j.rmed.2007.11.003 18083019

[B8] Chrystyn H Price D Not all asthma inhalers are the same: factors to consider when prescribing an inhaler Prim Care Respir J 2009 18 4 243 9 10.4104/pcrj.2009.00029 19513494 PMC6619362

[B9] Dolovich MB Ahrens RC Hess DR Anderson P Dhand R Rau JL Smaldone GC Guyatt G American College of Chest Physicians American College of Asthma, Allergy, and Immunology Device selection and outcomes of aerosol therapy: Evidence-based guidelines: American College of Chest Physicians/American College of Asthma, Allergy, and Immunology Chest 2005 127 1 335 71 10.1378/chest.127.1.335 15654001

[B10] Brocklebank D Ram F Wright J Barry P Cates C Davies L Douglas G Muers M Smith D White J Comparison of the effectiveness of inhaler devices in asthma and chronic obstructive airways disease: a systematic review of the literature Health Technol Assess 2001 5 26 1 149 11701099 10.3310/hta5260

[B11] Schulte M Osseiran K Betz R Wencker M Brand P Meyer T Haidl P Handling of and preferences for available dry powder inhaler systems by patients with asthma and COPD J Aerosol Med Pulm Drug Deliv 2008 21 4 321 8 10.1089/jamp.2007.0634 18823257

[B12] Molimard M Le Gros V Impact of patient-related factors on asthma control J Asthma 2008 45 2 109 13 10.1080/02770900701815727 18350401

[B13] Molimard M Raherison C Lignot S Depont F Abouelfath A Moore N Assessment of handling of inhaler devices in real life: an observational study in 3811 patients in primary care J Aerosol Med 2003 16 3 249 54 10.1089/089426803769017613 14572322

[B14] Bousquet J Poli G Acerbi D Monno R Ramael S Nollevaux F Systemic exposure and implications for lung deposition with an extra-fine hydrofluoroalkane beclometasone dipropionate/formoterol fixed combination Clin Pharmacokinet 2009 48 6 347 58 10.2165/00003088-200948060-00001 19650674

[B15] De Backer W Devolder A Poli G Acerbi D Monno R Herpich C Sommerer K Meyer T Mariotti F Lung Deposition of BDP/Formoterol HFA pMDI in Healthy Volunteers, Asthmatic, and COPD Patients J Aerosol Med Pulm Drug Deliv 2010 23 3 137 48 10.1089/jamp.2009.0772 20109122 PMC3123836

[B16] Huchon G Magnussen H Chuchalin A Dymek L Gonod FB Bousquet J Lung function and asthma control with beclomethasone and formoterol in a single inhaler Respir Med 2009 103 1 41 9 10.1016/j.rmed.2008.09.002 18977646

[B17] Fabbri LM Nicolini G Olivieri D Papi A Inhaled beclometasone dipropionate/formoterol extra-fine fixed combination in the treatment of asthma: evidence and future perspectives Expert Opin Pharmacother 2008 9 3 479 90 10.1517/14656566.9.3.479 18220498

[B18] Usmani OS Biddiscombe MF Barnes PJ Regional lung deposition and bronchodilator response as a function of beta_2_-agonist particle size Am J Respir Crit Care Med 2005 172 12 1497 504 10.1164/rccm.200410-1414OC 16192448

[B19] Leach CL Davidson PJ Hasselquist BE Boudreau RJ Influence of particle size and patient dosing technique on lung deposition of HFA-beclomethasone from a metered dose inhaler J Aerosol Med 2005 18 4 379 85 10.1089/jam.2005.18.379 16379614

[B20] Partridge MR Asthma: 1987-2007. What have we achieved and what are the persisting challenges? Prim Care Respir J 2007 16 3 145 8 10.3132/pcrj.2007.00039 17530142 PMC6634208

[B21] Miller MR Crapo R Hankinson J Brusasco V Burgos F Casaburi R Coates A Enright P van der Grinten CPM Gustafsson P Jensen R Johnson DC MacIntyre N McKay R Navajas D Pedersen OF Pellegrino R Viegi G Wanger J General considerations for lung function testing Eur Respir J 2005 26 153 161 10.1183/09031936.05.00034505 15994402

[B22] Papi A Paggiaro PL Nicolini G Vignola AM Fabbri LM Inhaled Combination Asthma Treatment versus SYmbicort TBH (ICAT SY) Study Group Beclomethasone/formoterol versus budesonide/formoterol combination therapy in asthma Eur Respir J 2007 29 4 682 9 10.1183/09031936.00095906 17107988

[B23] Papi A Paggiaro P Nicolini G Vignola AM Fabbri LM ICAT SE study group Beclomethasone/formoterol vs fluticasone/salmeterol inhaled combination in moderate to severe asthma Allergy 2007 62 10 1182 8 10.1111/j.1398-9995.2007.01493.x 17845589

[B24] Kraft M Djukanovic R Wilson S Holgate ST Martin RJ Alveolar tissue inflammation in asthma Am J Respir Crit Care Med 1996 154 5 1505 10 8912772 10.1164/ajrccm.154.5.8912772

[B25] Hamid Q Song Y Kotsimbos TC Minshall E Bai TR Hegele RG Hogg JC Inflammation of small airways in asthma J Allergy Clin Immunol 1997 100 1 44 51 10.1016/S0091-6749(97)70193-3 9257786

[B26] Glover W Chan HK Eberl S Daviskas E Verschuer J Effect of particle size of dry powder mannitol on the lung deposition in healthy volunteers Int J Pharm 2008 349 1-2 314 22 10.1016/j.ijpharm.2007.08.013 17904774

[B27] Zetterström O Buhl R Mellem H Perpiñá M Hedman J O'Neill S Ekström T Improved asthma control with budesonide/formoterol in a single inhaler, compared with budesonide alone Eur Respir J 2001 18 2 262 8 10.1183/09031936.01.00065801 11529282

[B28] Foster JM van Sonderen E Lee AJ Sanderman R Dijkstra A Postma DS van der Molen T A self-rating scale for patient-perceived side effects of inhaled corticosteroids Respir Res 2006 7 131 10.1186/1465-9921-7-131 17062139 PMC1637103

[B29] Peters SP Jones CA Haselkorn T Mink DR Valacer DJ Weiss ST Real-world Evaluation of Asthma Control and Treatment (REACT): findings from a national Web-based survey J Allergy Clin Immunol 2007 119 6 1454 61 10.1016/j.jaci.2007.03.022 17481716

